# A streamlined approach to classifying and tailoring implementation strategies: recommendations to speed the translation of research to practice

**DOI:** 10.1186/s43058-024-00606-8

**Published:** 2024-06-17

**Authors:** Jennifer Leeman, Catherine Rohweder, Jennifer Elston Lafata, Mary Wangen, Renee Ferrari, Christopher M. Shea, Alison Brenner, Isabel Roth, Oscar Fleming, Mark Toles

**Affiliations:** 1 School of Nursing, University of North Carolina at Chapel Hill, Chapel Hill, NC USA; 2 Lineberger Comprehensive Cancer Center, University of North Carolina at Chapel Hill, Chapel Hill, NC USA; 3 Center for Health Promotion and Disease Prevention, University of North Carolina at Chapel Hill, Chapel Hill, NC USA; 4 University of North Carolina at Chapel Hill, Center for Women’s Health Research, Chapel Hill, NC USA; 5 University of North Carolina at Chapel Hill, Eshelman School of Pharmacy, Chapel Hill, NC USA; 6 Gillings School of Global Public Health, University of North Carolina at Chapel Hill, Chapel Hill, NC USA; 7 School of Medicine, University of North Carolina at Chapel Hill, Chapel Hill, NC USA

**Keywords:** Implementation strategies, Implementation processes, Capacity building, Tailoring, Expert Recommendations for Implementing Change

## Abstract

**Background:**

Implementation science emerged from the recognized need to speed the translation of effective interventions into practice. In the US, the science has evolved to place an ever-increasing focus on implementation strategies. The long list of implementation strategies, terminology used to name strategies, and time required to tailor strategies all may contribute to delays in translating evidence-based interventions (EBIs) into practice. To speed EBI translation, we propose a streamlined approach to classifying and tailoring implementation strategies.

**Main text:**

A multidisciplinary team of eight scholars conducted an exercise to sort the Expert Recommendations for Implementing Change (ERIC) strategies into three classes: implementation processes (*n* = 25), capacity-building strategies (*n* = 20), and integration strategies (*n* = 28). Implementation processes comprise best practices that apply across EBIs and throughout the phases of implementation from exploration through sustainment (e.g., conduct local needs assessment). Capacity-building strategies target either general or EBI-specific knowledge and skills (e.g., conduct educational meetings). Integration strategies include “methods and techniques” that target barriers or facilitators to implementation of a specific EBI beyond those targeted by capacity building. Building on these three classes, the team collaboratively developed recommendations for a pragmatic, five-step approach that begins with the implementation processes and capacity-building strategies practice-settings are already using prior to tailoring integration strategies. A case study is provided to illustrate use of the five-step approach to tailor the strategies needed to implement a transitional care intervention in skilled nursing facilities.

**Conclusions:**

Our proposed approach streamlines the formative work required prior to implementing an EBI by building on practice partner preferences, expertise, and infrastructure while also making the most of prior research findings.

Contributions to the literature:

•We present a novel sorting of the Expert Recommendations for Implementing Change (ERIC) into three classes: implementation processes, capacity-building strategies, and integration strategies.
•Building on these three classes, we recommend a five-step approach to tailoring implementation strategies that leverages prior research findings together with practice partners’ preferences, expertise, and infrastructure.
•In our five-step approach, we recommend starting with the implementation processes and capacity-building strategies that practice partners are already using.
•Our approach has the potential to (a) reduce the formative work required to tailor implementation strategies, (b) remove barriers to research/practice partnerships, and (c) accelerate the translation of EBIs to practice.

## Background

The National Institutes of Health defines implementation research as “the scientific study of the use of strategies to adopt and integrate evidence-based health interventions into clinical and community settings to improve individual outcomes and benefit population health” [1]. Central to this definition is “the use of strategies”, commonly referred to as “implementation strategies”. Implementation strategies encompass a range of “methods and techniques used to enhance the adoption, implementation, and sustainability” of evidence-based interventions (EBIs) into clinical and community settings [2]. Implementation scholars have recommended multiple approaches that implementation researchers and practitioners (i.e., implementation planners) might use to select and tailor implementation strategies, including implementation mapping, concept mapping, and system dynamics modeling and [3–6]. Each of these approaches begins with practice-engaged formative work to identify the multilevel factors (i.e., determinants) that influence EBI implementation. Implementation planners then select strategies and tailor them to address barriers and facilitators to an EBI’s implementation at the level of the population, setting, community, or wider sociopolitical context [3, 5]. By tailoring to the needs of diverse contexts, implementation strategies have the potential to promote equitable implementation outcomes (e.g., reach, adoption, and fidelity) across populations and settings [7].

Although they offer many benefits, current approaches to tailoring implementation strategies also may slow the translation of EBIs into practice. Recent commentaries have highlighted the following challenges: 1) tailoring implementation strategies is often resource and time intensive, 2) lists of implementation strategies are long and complex, 3) in current approaches, implementation planners often prioritize their methods and perspectives over those of their practice partners [5, 8, 9].

### Tailoring implementation strategies is often resource and time intensive

Implementation planners may spend one or more years engaging community, patient, provider, and other practice partners in formative work to identify determinants and select implementation strategies [8]. This investment of time and resources may deter practice partners from collaborating on implementation studies. Community members, providers, and other practice partners are motivated by the desire to improve healthcare and health outcomes and may resist approaches that delay action to address pressing healthcare problems [10]. Finally, formative work may have high opportunity costs to the extent that investing in strategy tailoring for one EBI diverts time and energy from other health care problems.

Investing in extensive formative work to align strategies with determinants might be justified if there were evidence to support its value. However, empirical support is limited for the approaches currently used to tailor implementation strategies. In a study that involved 169 implementation researchers and practitioners, Waltz et al. (2019) reported extensive heterogeneity in recommendations for which of 73 implementation strategies would best address each of 39 implementation determinants [11]. Furthermore, determinants may vary across settings and rapidly changing environments, as occurred during the COVID-19 pandemic [8]. The ability to optimize implementation across contextual and temporal variations in determinants will require a more rapid and pragmatic approach to strategy tailoring. Balis and Houghtaling (2023) illustrate the challenge, by describing the difficulties experienced in their efforts to tailor strategies to implement nutrition and physical activity policy, systems, and environmental change interventions [12].

### Lists of implementation strategies are long and complex

Implementation planners and their academic and practice partners may be deterred by the length and complexity of current lists of implementation strategies [8]. Current lists may fail to include practice partners’ preferred methods for planning and implementing new interventions (e.g., methods from quality improvement, Six Sigma, program planning and evaluation) [13]. Even when methods align, the terminology used to name those methods often differs between research and practice.

### Researchers prioritize their methods and perspectives over those of their practice partners’ [8]

When implementation researchers begin with their preferred list of strategies rather than their practice partners’ preferences, they risk creating barriers rather than bridges. Miller et al. summarize the problem as an “overall approach that disrespects and undervalues primary care as a coproducer of knowledge and inadvertently bullies practices into conforming to goals they did not choose” [9].

We argue for a more pragmatic approach that reclassifies implementation strategies and promotes more purposeful alignment with the methods practice partners use to implement change. Previous scholars have developed multiple lists and classifications of implementation strategies [3]. One of the most widely used lists is the Expert Recommendations for Implementing Change (ERIC) compendium of 73 strategies [14]. Waltz et al. further classified the ERIC strategies into broad “conceptually relevant groupings” [15]. Similarly, Mazza et al. classified the Cochrane Effective Practice and Organization of Care (EPOC) taxonomy of strategies into four broad domains [16]. To create the Behavior Change Wheel, Michie et al. aligned strategies with their functions, in other words with their theory-derived methods [17]. While all of these efforts have value, none has attempted to classify strategies according to 1) those that require formative work to identify EBI-specific barriers versus 2) those that build on the methods that practice partners are already using.

## Main text

We recognize the value of conducting formative work prior to selecting and tailoring implementation strategies. However, we argue for reducing the investment in formative work by starting with the strategies that practice partners are already using. We identify three classes of implementation strategies and propose a pragmatic, five-step approach that implementation planners might use to select and tailor strategies within these three classes. We illustrate the five-step approach with a case study from two authors’ research on transitional care interventions in skilled nursing facilities.

### Three classes of implementation strategies

We contend that not all implementation strategies need to be tailored. Some strategies involve best practices that apply across most EBIs and may not require tailoring to the implementation context. To generate classes of implementation strategies, we conducted a sorting exercise with the 73 strategies in the ERIC taxonomy. Our approach to sorting strategies builds on the classification system created by Leeman et al., [18] which identifies five classes that differ according to the a) level of “actor” executing the strategy (level of the delivery system or external support system) and b) type of determinants targeted (EBI agnostic versus specific). We were most interested in distinguishing between strategies that were EBI agnostic versus EBI specific and less interested in the level of actor. For this reason, we initially combined the two classes that were EBI agnostic: capacity-building and implementation processes. We also combined the two that were EBI specific: scale-up strategies and integration strategies. Last, we decided not to include the fifth class, dissemination strategies, because it comprises strategies that are not included in the ERIC taxonomy. This left two classes of strategies: implementation processes and integration strategies. *Implementation processes* apply across EBIs (i.e., are EBI agnostic) and comprise the activities involved in planning, selecting, implementing, and sustaining an EBI. *Integration strategies* are “actions that target factors contributing to or impeding the optimal integration of a specific EBI into practice” [18].

A team of eight scholars completed the sorting. Team members were experts in implementation research and/or implementation practice and represented schools of nursing, pharmacy, public health, and medicine. We started by asking team members to independently sort the 73 strategies in the ERIC compendium [14] into two classes: implementation processes or integration strategies. In the first round of sorting, the team achieved a high level of agreement on 35 items in the ERIC compendium, with at least 7 of 8 agreeing on the same classification for each item. Consensus meetings were subsequently held to classify the remaining 38 items. In the first meeting, the team reached consensus on 13 items that could be either EBI agnostic or EBI specific and determined they were best classified as capacity-building strategies. We therefore added a third classification, revising the Leeman definition of capacity-building to encompass EBI-specific in addition to EBI-general capacity [18]. We defined capacity-building strategies as strategies that target individual-level capacity (knowledge, skills) to select, adapt, and/or implement EBIs generally or to implement a specific EBI. The group met two more times to classify the remaining 25 strategies. In the initial sorting, levels of consensus on these strategies varied, with six of eight (75%) team members agreeing on 11 of the strategies, five of eight agreeing on five strategies, and fewer than five agreeing on the remaining nine strategies. During consensus discussions, the team identified challenges when sorting those ERIC strategies that have broad or multi-component definitions. For example, the strategy “model and simulate change” is broadly defined as “Model or simulate the change that will be implemented prior to implementation”, and the definition for the strategy, “provide clinical supervision”, describes multiple components, including both supervision and training [14].

Through iterative discussions, the team reached consensus on the classification of 25 implementation processes (Table 1), 20 capacity-building strategies (Table 2), and 28 integration strategies (Table 3).

**Table 1:** Implementation processes: name and definition [14]

Strategy Name	Definition
Assess for readiness and identify barriers and facilitators	Assess various aspects of an organization to determine its degree of readiness to implement, barriers that may impede implementation, and strengths that can be used in the implementation effort
Conduct cyclical small tests of change	Implement changes in a cyclical fashion using small tests of change before taking changes system-wide. Tests of change benefit from systematic measurement, and results of the tests of change are studied for insights on how to do better. This process continues serially over time, and refinement is added with each cycle
Conduct local consensus discussions	Include local providers and other stakeholders in discussions that address whether the chosen problem is important and whether the clinical innovation to address it is appropriate
Conduct local needs assessment	Collect and analyze data related to the need for the innovation
Develop a formal implementation blueprint	Develop a formal implementation blueprint that includes all goals and strategies. The blueprint should include the following: 1) aim/purpose of the implementation; 2) scope of the change (*e.g.*, what organizational units are affected); 3) timeframe and milestones; and 4) appropriate performance/progress measures. Use and update this plan to guide the implementation effort over time
Develop an implementation glossary	Develop and distribute a list of terms describing the innovation, implementation, and stakeholders in the organizational change
Develop and implement tools for quality monitoring	Develop, test, and introduce into quality-monitoring systems the right input—the appropriate language, protocols, algorithms, standards, and measures (of processes, patient/consumer outcomes, and implementation outcomes) that are often specific to the innovation being implemented
Develop and organize quality monitoring systems	Develop and organize systems and procedures that monitor clinical processes and/or outcomes for the purpose of quality assurance and improvement
Identify and prepare champions	Identify and prepare individuals who dedicate themselves to supporting, marketing, and driving through an implementation, overcoming indifference or resistance that the intervention may provoke in an organization
Identify early adopters	Identify early adopters at the local site to learn from their experiences with the practice innovation
Inform local opinion leaders	Inform providers identified by colleagues as opinion leaders or “educationally influential” about the clinical innovation in the hopes that they will influence colleagues to adopt it
Intervene with patients/consumers to enhance uptake and adherence	Develop strategies with patients to encourage and problem solve around adherence
Involve executive boards	Involve existing governing structures (*e.g.*, boards of directors, medical staff boards of governance) in the implementation effort, including the review of data on implementation processes
Involve patients/ consumers and family members	Engage or include patients/consumers and families in the implementation effort
Model and simulate change	Model or simulate the change that will be implemented prior to implementation
Obtain formal commitments	Obtain written commitments from key partners that state what they will do to implement the innovation
Obtain and use patients/consumers and family feedback	Develop strategies to increase patient/consumer and family feedback on the implementation effort

**Table 2:** Capacity-building strategies: name and definition [14]

Strategy Name	Definition
Capture and share local knowledge	Capture local knowledge from implementation sites on how implementers and clinicians made something work in their setting and then share it with other sites
Centralize technical assistance	Develop and use a centralized system to deliver technical assistance focused on implementation issues
Conduct educational meetings	Hold meetings targeted toward different stakeholder groups (*e.g.*, providers, administrators, other organizational stakeholders, and community, patient/consumer, and family stakeholders) to teach them about the clinical innovation
Conduct educational outreach visits	Have a trained person meet with providers in their practice settings to educate providers about the clinical innovation with the intent of changing the provider’s practice
Conduct ongoing training	Plan for and conduct training in the clinical innovation in an ongoing way
Create a learning collaborative	Facilitate the formation of groups of providers or provider organizations and foster a collaborative learning environment to improve implementation of the clinical innovation
Develop academic partnerships	Partner with a university or academic unit for the purposes of shared training and bringing research skills to an implementation project
Develop educational materials	Develop and format manuals, toolkits, and other supporting materials in ways that make it easier for stakeholders to learn about the innovation and for clinicians to learn how to deliver the clinical innovation
Distribute educational materials	Distribute educational materials (including guidelines, manuals, and toolkits) in person, by mail, and/or electronically
Facilitation	A process of interactive problem solving and support that occurs in a context of a recognized need for improvement and a supportive interpersonal relationship
Make training dynamic	Vary the information delivery methods to cater to different learning styles and work contexts, and shape the training in the innovation to be interactive
Provide local technical assistance	Develop and use a system to deliver technical assistance focused on implementation issues using local personnel
Provide ongoing consultation	Provide ongoing consultation with one or more experts in the strategies used to support implementing the innovation
Shadow other experts	Provide ways for key individuals to directly observe experienced people engage with or use the targeted practice change/innovation
Start a dissemination organization	Identify or start a separate organization that is responsible for disseminating the clinical innovation. It could be a for-profit or non-profit organization
Use an implementation advisor	Seek guidance from experts in implementation
Use data experts	Involve, hire, and/or consult experts to inform management on the use of data generated by implementation efforts
Use train-the-trainer strategies	Train designated clinicians or organizations to train others in the clinical innovation
Visit other sites	Visit sites where a similar implementation effort has been considered successful
Work with educational institutions	Encourage educational institutions to train clinicians in the innovation

**Table 3:** Integration strategies: name and definition (14)

Strategy name	Definition
Access new funding	Access new or existing money to facilitate the implementation
Alter incentive/ allowance structures	Work to incentivize the adoption and implementation of the clinical innovation
Alter patient/consumer fees	Create fee structures where patients/consumers pay less for preferred treatments (the clinical innovation) and more for less-preferred treatments
Audit and provide feedback	Collect and summarize clinical performance data over a specified time period and give it to clinicians and administrators to monitor, evaluate, and modify provider behavior
Build a coalition	Recruit and cultivate relationships with partners in the implementation effort
Change accreditation or membership requirements	Strive to alter accreditation standards so that they require or encourage use of the clinical innovation. Work to alter membership organization requirements so that those who want to affiliate with the organization are encouraged or required to use the clinical innovation
Change liability laws	Participate in liability reform efforts that make clinicians more willing to deliver the clinical innovation
Change physical structure and equipment	Evaluate current configurations and adapt, as needed, the physical structure and/or equipment (*e.g.*, changing the layout of a room, adding equipment) to best accommodate the targeted innovation
Change record systems	Change records systems to allow better assessment of implementation or clinical outcomes
Change service sites	Change the location of clinical service sites to increase access
Create new clinical teams	Change who serves on the clinical team, adding different disciplines and different skills to make it more likely that the clinical innovation is delivered (or is more successfully delivered)
Create or change credentialing and/or licensure standards	Create an organization that certifies clinicians in the innovation or encourage an existing organization to do so. Change governmental professional certification or licensure requirements to include delivering the innovation. Work to alter continuing education requirements to shape professional practice toward the innovation
Develop disincentives	Provide financial disincentives for failure to implement or use the clinical innovations
Develop resource sharing agreements	Develop partnerships with organizations that have resources needed to implement the innovation
Facilitate relay of clinical data to providers	Provide as close to real-time data as possible about key measures of process/outcomes using integrated modes/channels of communication in a way that promotes use of the targeted innovation
Fund and contract for the clinical innovation	Governments and other payers of services issue requests for proposals to deliver the innovation, use contracting processes to motivate providers to deliver the clinical innovation, and develop new funding formulas that make it more likely that providers will deliver the innovation
Increase demand	Attempt to influence the market for the clinical innovation to increase competition intensity and to increase the maturity of the market for the clinical innovation
Make billing easier	Make it easier to bill for the clinical innovation
Mandate change	Have leadership declare the priority of the innovation and their determination to have it implemented
Place innovation on fee for service lists/formularies	Work to place the clinical innovation on lists of actions for which providers can be reimbursed (*e.g.*, a drug is placed on a formulary, a procedure is now reimbursable)
Prepare patients/consumers to be active participants	Prepare patients/consumers to be active in their care, to ask questions, and specifically to inquire about care guidelines, the evidence behind clinical decisions, or about available evidence-supported treatments
Provide clinical supervision	Provide clinicians with ongoing supervision focusing on the innovation. Provide training for clinical supervisors who will supervise clinicians who provide the innovation
Remind clinicians	Develop reminder systems designed to help clinicians to recall information and/or prompt them to use the clinical innovation
Revise professional roles	Shift and revise roles among professionals who provide care, and redesign job characteristics
Use capitated payments	Pay providers or care systems a set amount per patient/consumer for delivering clinical care
Use data warehousing techniques	Integrate clinical records across facilities and organizations to facilitate implementation across systems
Use mass media	Use media to reach large numbers of people to spread the word about the clinical innovation
Use other payment schemes	Introduce payment approaches (in a catch-all category)

### Recommendations for a new approach

**Fig. 1:**
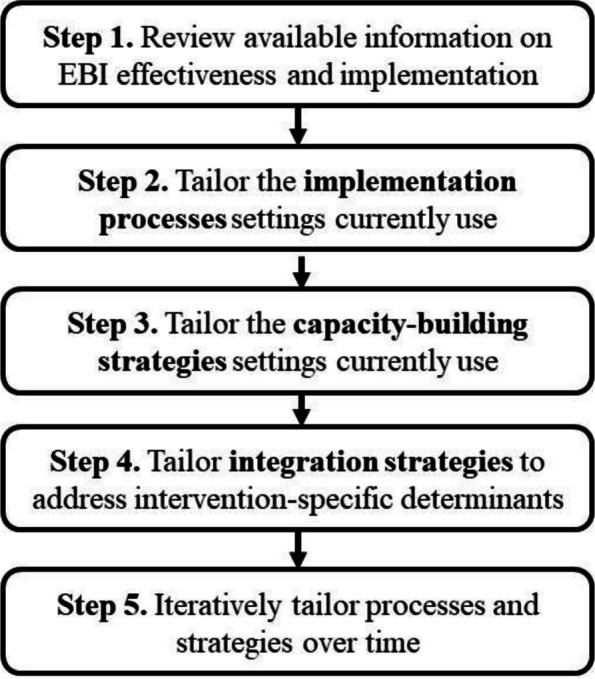
Five-step approach to tailoring implementation strategies

Below, we propose a pragmatic, five-step approach for implementation planners to use when selecting and tailoring implementation strategies (Fig. 1). Implementation planners may apply this approach in partnership with intervention developers and with clinical or public health systems or settings with access to expertise in the intervention being implemented. Our recommendations build on the work of multiple implementation scientists over many years (e.g., [4, 5]). We add to prior work by highlighting how our re-conceptualization of implementation strategies has potential to speed the translation of research to practice.

#### Step 1. Review available information on EBI effectiveness and implementation

Once an implementation planner knows what EBI will be implemented, we recommend that they review reports from prior research and practice-based evaluations of the EBI. If possible, they might talk with a) researchers who have studied the EBI’s effectiveness and/or implementation and b) decision makers, providers, and staff who have implemented the EBI into real-world practice settings. The types of information resulting from these discussion will include evidence on effectiveness and may also include lists of potential barriers and facilitators (i.e., determinants), materials to support implementation (e.g., intervention protocols), and evidence on variations in implementation across different populations and settings [19].

As they review the assembled information, implementation planners should consider the EBI’s level of complexity and uncertainty. These two characteristics of EBIs are especially important in determining the level of investment required to tailor implementation strategies [20]. EBI complexity refers to the extent to which implementing and delivering an EBI is intricate or complicated. Several factors increase an EBI’s complexity, including the number of socioecological levels targeted, the diversity and interdependence of implementers (e.g., from different organizations and/or disciplines), number of EBI components, and the duration and number of contacts required for EBI delivery [20, 21]. Uncertainty refers to limits on available information about how to implement the EBI or how implementation may vary across contexts [20]. Some EBIs provide guidance on implementation, which may include training curricula, delivery protocols, and intervention materials as part of an “intervention package” [19]. For other EBIs, limited information is available on implementation or implementation is specific to each context (e.g., changes to local policies and environments) [22]. Selecting implementation processes and strategies for a relatively low complexity, low uncertainty EBI (e.g., patient reminders for colorectal cancer screening) will require less investment in formative work than for a complex, and/or high uncertainty EBI (e.g., multi-component interventions to reduce tobacco marketing at the point of sale).

#### Step 2. Identify and tailor the implementation processes that practice settings currently use

Implementation processes represent best practices for implementation and, as such, are relevant to most implementation initiatives [23]. These processes encompass the activities and infrastructure required to plan, execute, and evaluate EBI implementation [24] across all stages, starting with initial exploration and continuing through sustainment [25]. Examples of implementation processes include “assessing barriers and facilitators” and “convening implementation teams.” To select implementation processes, begin by identifying the processes practice settings are already using to implement change, such as quality improvement, Six Sigma, program planning, or other processes [13]. Incorporating these processes into an implementation plan may require only minimal tailoring.

Once you have identified the processes that practice settings are using, ground implementation in those processes and align them with the EBI’s complexity and uncertainty. For example, if the EBI has a relatively low level of complexity and uncertainty, implementation may involve processes such as creating a small, short-term quality improvement (QI) team to implement the EBI coupled with a system for ongoing monitoring and evaluation. For EBIs with high levels of complexity and uncertainty, practice partners may engage in more involved processes to map current and future process flow diagrams, conduct root cause analyses, and complete plan-do-study-act cycles to iteratively test different options for implementing the EBI into routine practice [13].

#### Step 3. Identify and tailor the capacity-building strategies that practice settings currently use

Capacity-building strategies target individual-level knowledge and skills. These strategies may build capacity to engage in implementation processes (general capacity) and/or to deliver a specific EBI (EBI-specific capacity) [26]. Examples of capacity-building strategies include educational meetings, facilitation, and ongoing training. These strategies may be delivered by individuals within or external to the implementation setting. Examples of organizations that deliver capacity-building strategies include the American Cancer Society, State Health Departments, and a range of other entities that routinely provide training, technical assistance, and other supports to practice settings.

Moreover, when selecting capacity-building strategies, consider those that the practice setting and/or external partners prefer and have the expertise and infrastructure to support. Once the strategy is selected, some tailoring is often required to address gaps in the intended audiences’ knowledge and skills. This may include addressing gaps related to general (e.g., best practices for implementing change) and/or EBI-specific capacity (e.g., how to deliver or implement a specific intervention). Assessment of capacity typically involves interviews or surveys of individuals at participating sites, with a focus on their expertise and prior experience related to selected processes for implementing change or to a specific EBI. Assessment findings then can be used to tailor the content and dose of capacity-building strategies.

Many resources are available for use in building general capacity, for example the Institute for Healthcare Improvement’s Open School [27] or the Cancer Prevention and Control Research Network training opportunities [28]. To build EBI-specific capacity, where possible, start with materials and other resources developed in prior work to test and/or implement the EBI. At the end of intervention testing, researchers often have intervention protocols, training curriculum, and other guidance related to implementation.

#### Step 4. Tailor integration strategies to address EBI-specific implementation determinants

By definition, integration strategies “target factors contributing to or impeding the optimal integration of a specific EBI into practice” [11]. Therefore, tailoring integration strategies starts with considering factors (i.e., determinants) that contribute to or impede integration [3]. Review of existing data from studies of an EBI (Step 1) often identifies factors that influence implementation (i.e., determinants). Literature reviews of efforts to implement similar EBIs also may yield findings on determinants. If available data on determinants are limited or implementation is in a novel setting, consider one or more of the emerging approaches to rapidly identify determinants [8, 29].

Once determinants are identified, strategies are then selected and tailored to target those determinants. Rather than linking determinants directly to implementation strategies, Fernandez and colleagues recommend linking determinants to theory-derived methods [4]. Multiple behavioral change and organizational theories are available that hypothesize methods for addressing determinants [17, 30]. To illustrate, believing that others view a behavior as desirable (i.e., subjective norms) is a commonly identified, theory-based, determinant of EBI implementation. Behavior change theory proposes that seeing respected peers model the behavior is one method for influencing subjective norms. Linking the determinant to a theory-derived method then guides the selection of implementation strategies from among those that may include modeling (e.g., provide audit & feedback, shadow other experts, visit other sites). Once an implementation strategy is selected, it may then be tailored to fit the EBI and context as well as to comply with parameters specified by the theory. For example, the person modeling the behavior should be someone respected by the clinicians (or relevant others) within the practice setting.

#### Step 5. Iteratively tailor implementation strategies overtime

Completion of the first four steps will yield a set of implementation strategies for a specific EBI. The deployment of those strategies provides an opportunity to collect data to guide further tailoring of strategies. Widely-used methods for evaluating implementation include periodic reflection and sequential explanatory mixed-methods. In the former, an evaluator meets periodically with implementers to understand how and why they are adapting implementation strategies and/or to reflect on aspects of implementation that are working well or that present opportunities for improvement [31, 32]. In the latter method, evaluators review data on implementation outcomes (e.g., reach, fidelity) and then interview implementers to understand the reasons for gaps or variations in those outcomes [33]. Evaluation may include examination of whether implementers applied processes as intended, how they adapted them, and factors that influenced when and how they applied implementation processes. It may also include an assessment of implementers’ capacity, with the goal of identifying continuing gaps in knowledge and skills. Finally, evaluation may include an exploration of unanticipated barriers to implementation at the level of the delivery setting or its wider context. Each time an EBI is implemented, there is an opportunity for further learning and refinement of all three classes of implementation strategies.

### Case example

To illustrate, we describe application of the five proposed steps to the Connect-Home Transitional Care Intervention (Connect-Home) [34]. Connect-Home is delivered by multidisciplinary care teams (social worker, nurse, rehabilitation specialists) in skilled nursing facilities (SNFs) to prepare older adults and their caregivers for the transition to home. Core components of Connect-Home include protocols for (a) convening an interdisciplinary planning meeting with the older adult and their caregiver, (b) creating an interdisciplinary post discharge plan of care, (c) transitioning care to community providers, and (d) providing in-person or telephone-based post-discharge support [35]. Below we describe the steps an implementation researcher took to develop Connect-Home implementation strategies, in close partnership with the developer of the Connect-Home intervention.

#### Step 1. Review available information on EBI effectiveness and implementation

Plans for Connect-Home implementation started with a review of findings and products from the formative work done to develop Connect-Home. Formative work included qualitative case studies of transitional care in SNFs [36], systematic review of the literature [37], and findings from feasibility studies of the Connect-Home Intervention [38].

Connect-Home is a complex intervention that involves interaction among a multiple disciplinary team over an extended period of time. Guidance on Connect-Home implementation is included in the intervention package. However, SNFs had only applied this guidance within the context of research on Connect-Home efficacy, and little was known about how implementation would work in the less controlled, lower resourced context required for broadscale implementation. Implementing EBIs in SNFs is particularly challenging due to high staff turnover rates and limited infrastructure to support implementation [39, 40].

#### Step 2. Identify and tailor the implementation processes that practice settings currently use

To plan for implementation, the Connect-Home developer partnered with an implementation researcher and with representatives from a national chain of SNFs [41]. This planning team knew that federal mandates require SNFs to implement Quality Assurance and Performance Improvement (QAPI) programs. After reflecting on the experience of research and practice partners, the planning team determined that SNF staff have at least some familiarity with two implementation processes: QI teams and Plan-Do-Study-Act or PDSA cycles (equivalent to ERIC strategies: “organize clinician implementation team meetings” and “conduct small cyclical tests of change”). The planning team then tailored these two processes for Connect-Home. Specifically, they determined that QI teams needed to include representatives from each of the disciplines involved in Connect-Home delivery and developed protocols for teams to complete three cycles of PDSAs.

#### Step 3. Identify and tailor the capacity-building strategies that practice settings currently use

The national SNF chain had experience using learning collaboratives as a strategy to introduce SNFs to new interventions [41]. Therefore, the planning team embedded capacity building within a learning collaborative model (ERIC strategy “create a learning collaborative”). They invited two representatives from each SNF’s QI team to attend two in-person collaborative meetings. The first collaborative meeting provided an overview of Connect-Home and training on how to use implementation processes (i.e., QI teams and PDSA cycles) to implement Connect-Home within a SNF. At the second meeting, participants shared their experience implementing Connect-Home and successful strategies for overcoming barriers. The learning collaborative was supplemented with strategies developed during formative research (Step 1), including, an intervention manual and on-site training on intervention delivery (ERIC strategies: “distribute educational materials” and “conduct educational meetings”). During feasibility testing of Connect-Home efficacy, Connect-Home researchers had success providing monthly in-person consultations on intervention delivery to SNF staff. For implementation, the planning team modified consultation to enhance its scalability and include guidance on implementation. The team replaced in-person consultation with monthly web-conferenced coaching sessions to QI teams (ERIC: “facilitation”). During these monthly sessions, QI teams were coached through the completion of three PDSA cycles to iteratively improve Connect-Home implementation over time.

#### Step 4. Tailor integration strategies to address determinants

The formative work involved in developing Connect-Home identified many determinants of high-quality transitional care. To implement Connect-Home, the planning team selected and tailored strategies to target these determinants. For example, the team changed record systems (an ERIC strategy) to target interdisciplinary communication. Each participating SNF was asked to embed the Connect-Home plan of care template within their electronic health record. Then, every three months, data were pulled from the SNF EHR systems to assess each care team member’s fidelity to protocols for completing their sections of the plan of care. These data were then provided to the implementation team to aid in identifying areas of focus in their PDSA cycles (ERIC strategy “audit and provide feedback”).

#### Step 5. Iteratively tailor implementation strategies over time

The planning team received two grants to implement Connect-Home in six states [34]. With each round of implementation, the team used evaluation data to further tailor implementation strategies with the goal optimizing intervention reach, fidelity, and acceptability over time.

## Conclusions

In this paper, we propose three classes of implementation strategies as a first step toward improving communication among implementation planners and their academic and practice partners. Rather than engaging partners in a discussion of 73 strategies, we organize the strategies into three separate classes. To streamline the process of tailoring strategies, we propose a five-step approach that builds on these three classes. We recommend starting with a review of what is already known about the EBI and its implementation. This review provides information that is foundational to tailoring strategies, including an understanding of the EBI (i.e., complexity and uncertainty), known determinants of its implementation, and guidance available to support its implementation. We then recommend starting with the processes that partners are already using to implement changes to practice. Building on partners’ strengths is key to building trust and speeding translation of EBIs into practice; thus, in working with partners, we further recommend using terminology that is familiar to partners. For example, use the term “PDSA cycle,” a QI term familiar to practice partners, instead of the ERIC term—“conduct cyclical small tests of change.” We recognize the value of using standardized language (e.g., ERIC terminology) to report findings as it aids efforts to synthesize the findings needed to build the evidence base for implementation strategies. For this reason, we recommend that researchers use terms that are most familiar to their practice partners during implementation and then align those terms with standardized terminology when they report their findings.

As illustrated in our case study, the five-step approach we propose may still involve a substantial investment of time and resources. Nonetheless, we contend that this approach has the potential to speed implementation for at least some EBIs and generate efficiencies in how strategies are selected. This approach may expedite implementation by tailoring strategies contingent on an EBIs’ level of complexity and uncertainty. Furthermore, this approach generates efficiencies by 1) prioritizing strategies that are already supported within the practice setting and 2) taking advantage of findings from all stages of the research process. In addition to improving the efficiency of strategy selection, this approach has potential to reduce the burden to providers and staff resulting from the intensive formative work involved in some approaches to strategy tailoring.

We are not the first authors to sort ERIC strategies into classes [16, 42]. Our innovation was in sorting them according to whether strategies addressed barriers to implementation of a specific EBI or were more generally applicable. Further work is needed to refine the three proposed classes of implementation strategies. A potential next step is to incorporate other compendia of strategies and processes. For example, the Quality Implementation Framework may provide a starting point for refining the list of implementation processes [23]. Further research also is needed to compare the proposed approach to other approaches to selecting and tailoring implementation strategies. This research might evaluate differences in practice partners’ (1) perceptions of implementation strategies (acceptability, feasibility), (2) fidelity to implementation strategies, (3) implementation costs, and (4) adoption and sustainment of the EBI.

In a recent critique of implementation science, Biedas and colleagues note, “Our implementation strategies and processes were too complex and not well matched to partner needs” [8]. We begin to address this critique through our approach to strategy classification, selection, and tailoring. Our proposed approach streamlines the formative work prior to implementing an EBI by building on practice partner preferences, expertise, and infrastructure while also making the most of prior research findings.

## Data Availability

Not Applicable.

## Abbreviations

EBI: Evidence-based intervention
HER: Electronic Health Record
ERIC: Expert Recommendations for Implementing Change
PDSA: Plan, Do, Study, Act
QAPI: Quality Assurance and Performance Improvement
QI: Quality Improvement
SNF: Skilled Nursing Facility
